# Food-Derived Uremic Toxins in Chronic Kidney Disease

**DOI:** 10.3390/toxins15020116

**Published:** 2023-02-01

**Authors:** Mara Lauriola, Ricard Farré, Pieter Evenepoel, Saskia Adriana Overbeek, Björn Meijers

**Affiliations:** 1 Laboratory of Nephrology and Renal Transplantation, Department of Microbiology, Immunology and Transplantation, KU Leuven, 3000 Leuven, Belgium; 2 Department of Nephrology and Renal Transplantation, University Hospitals Leuven, 3000 Leuven, Belgium; 3 Translational Research Center for Gastrointestinal Disorders (TARGID), Department of Chronic Diseases and Metabolism (CHROMETA), KU Leuven, 3000 Leuven, Belgium; 4 Danone Nutricia Research, 3584 Utrecht, The Netherlands

**Keywords:** uremic toxins, chronic kidney disease, metabolism, diet, nutrition, food

## Abstract

Patients with chronic kidney disease (CKD) have a higher cardiovascular risk compared to the average population, and this is partially due to the plasma accumulation of solutes known as uremic toxins. The binding of some solutes to plasma proteins complicates their removal via conventional therapies, e.g., hemodialysis. Protein-bound uremic toxins originate either from endogenous production, diet, microbial metabolism, or the environment. Although the impact of diet on uremic toxicity in CKD is difficult to quantify, nutrient intake plays an important role. Indeed, most uremic toxins are gut-derived compounds. They include Maillard reaction products, hippurates, indoles, phenols, and polyamines, among others. In this review, we summarize the findings concerning foods and dietary components as sources of uremic toxins or their precursors. We then discuss their endogenous metabolism via human enzyme reactions or gut microbial fermentation. Lastly, we present potential dietary strategies found to be efficacious or promising in lowering uremic toxins plasma levels. Aligned with current nutritional guidelines for CKD, a low-protein diet with increased fiber consumption and limited processed foods seems to be an effective treatment against uremic toxins accumulation.

## 1. Introduction

Chronic kidney disease (CKD) is estimated to affect around 846 million people in the world in 2022 [1]. Patients with CKD have a higher cardiovascular risk compared to the average population, and cardiovascular events represent the leading cause of death among these patients [2]. Retention of organic solutes is considered an important driving factor for the increased cardiovascular risk [3]. These solutes can be defined as uremic toxins when they meet specific criteria: their level must be increased in uremia; there must be a clear relationship with clinical manifestations; and these manifestations must show improvement when their levels decrease and lead to the deterioration of uremic symptoms upon administration of this molecule [4].

Several classifications of uremic retention solutes have been proposed [4]. Uremic toxins can be categorized according to their molecular size as small compounds (<500 Da) or medium compounds (molecular weight > 500 Da) and according to their binding capacity as water-soluble compounds or protein-bound toxins. The latter are considered challenging because of the difficulty in removing them from the systemic circulation via dialysis [3]. This is due to their high binding capacity to circulating proteins, such as albumin [5]. Among protein-bound uremic toxins, we can identify those generated within the human body, those derived from diet, and those originated from the external environment, such as exposure to bisphenol A [6,7]. Twenty-five protein-bound uremic toxins have been described in CKD [8]. The role of the gut microbiome as an important source of these metabolites is well recognized and is covered by several excellent reviews [9,10,11,12]. To what extent dietary intake plays a role is often not discussed in detail.

In this review, we focus on food-derived protein-bound uremic toxins, describing how these and their precursors are metabolized and how diet modifications may influence their plasma level. Table 1 lists protein-bound uremic toxins and their dietary origin. Protein-bound uremic toxins generated after gut microbial metabolism of dietary compounds will just be briefly mentioned since they have already been reviewed elsewhere [10,12]. 

## 2. Protein-Bound Uremic Toxins

**Figure 1:**
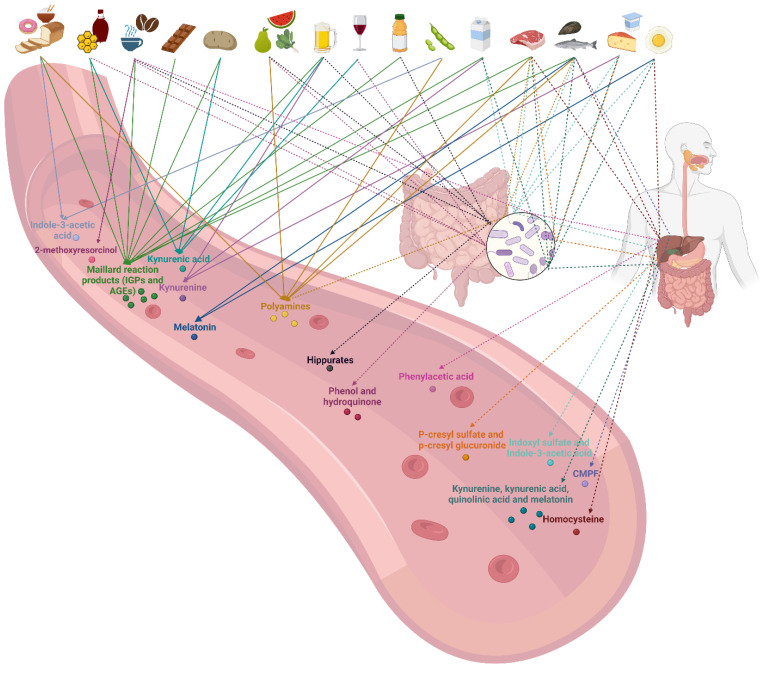
Overview of foods as sources of protein-bound uremic toxins. The dash lines indicate the metabolization of the precursors of protein-bound uremic toxins by the gut microbiome or by the host. IGPs = intermediate glycation products; AGEs = advanced glycation products; CMPF = 3-Carboxy-4-methyl-5-propyl-2-furanpropionate. Created with BioRender.com.

Maillard reaction products, hippurates, indoles, phenols, and polyamines have been described as protein-bound uremic toxins (PBUTs). Difficulties are encountered when trying to classify PBUTs according to their dietary origin because of the interaction of nutrients and because of the multiple precursors that can lead to the formation of each of these toxins during digestion or gut microbial fermentation. Figure 1 shows how foods we consume on a daily basis contribute directly or indirectly to the plasma retention of uremic toxins. Below, we discuss which derivatives of dietary carbohydrates, proteins, lipids, and micronutrients are the precursors of these PBUTs and how diet can contribute to their plasma retention. 

## 3. Carbohydrates

Dietary carbohydrates can be classified according to their degree of polymerization into sugars, including monosaccharides, disaccharides, polyols, oligosaccharides, and polysaccharides [44]. Due to their role in CKD, we focus on reducing sugars, i.e., sugars that, because of their aldehyde or ketone group, act as reducing agents in basic solutions. Glucose, fructose, galactose, lactose, and maltose are reducing sugars and can be considered the precursors of some PBUTs. In fact, they participate in the so-called Maillard reactions, i.e., chemical reactions between reducing sugars and amino acids that occur when cooking/baking/roasting food at high temperatures. These reactions confer browned food its distinctive flavor (cookies, biscuits, bread, fried dumplings, and toasted marshmallow) [45]. Products of Maillard reactions considered as uremic toxins include both intermediate glycation products (IGPs), i.e., fructoselysine, 3-deoxyglucosone, glyoxal, and methylglyoxal, and the more stable advanced glycation end-products (AGEs), i.e., Nε-carboxymethyllysine (CML), Nε-carboxyethyllysine (CEL), and and pentosidine [4]. 

Precautions can be taken to lower the amount of reducing sugars in foods. For instance, storing potatoes at temperatures above 8 °C helps to decrease the content of reducing sugars [44]. Using other cooking methods, such as steaming or boiling, and applying soaking or blanching before cooking can also diminish reducing sugars and, thereby, the formation of Maillard reaction products.

### 3.1. Maillard Reaction Products

Maillard reactions start with the formation of an unstable Schiff base that undergoes spontaneous rearrangement to form ketoamines, also known as Amadori products [45]. From the degradation of Amadori products, furfurals, reductones, and fragmentation products are generated. When the reaction goes further, these compounds start to condense without the need for other amino groups. Ultimately, reactions between these intermediate products with amino compounds lead to the formation of advanced glycation end-products (AGEs), also called melanoidins in food science. In general, Maillard reactions, also called non-enzymatic browning, can take place both in foods and within the human body. Indeed, glycation of body proteins, i.e., the reaction between glucose or its derived products with amines, amino acids, peptides, and proteins, can be seen as the first step of this series of reactions. While a low percentage of glycated proteins within the human body is considered physiological, several studies have confirmed that conditions of diabetes and uremia present higher plasma levels of IGPs and AGEs [45,46,47]. 

The mechanisms promoting the accumulation of these products are not yet fully elucidated. It is clear that despite a great contribution to the endogenous formation of these compounds, diet-derived IGPs and AGEs play an important role in CKD progression [46,48,49,50,51], especially outside of diabetes [52]. Uribarri et al. found a correlation between serum levels of AGEs with dietary AGE intake based on a 3-day food diary or dietary questionnaires in a cohort of 189 patients on dialysis [46]. The same authors found that nondiabetic CKD patients on peritoneal dialysis who were asked to follow a high-AGE diet for four weeks showed a higher plasma retention of IGPs and AGEs compared to those on a low-AGE diet [50]. Interestingly, a study in rats showed that the administration of a thermolyzed diet led to a sustained increased plasma level of Maillard reaction products during the intervention [51]. 

#### 3.1.1. Intermediate Glycation Products

##### Fructoselysine

Fructoselysine (FL), also called Nε -fructosyl-lysine, is the Amadori product of the amino acid lysine when the reducing sugar is glucose. FL is one of the most common Maillard reaction products present in processed foods, such as pasteurized milk, pasta, chocolate, cereals, and carbonated soft drinks [13]. Importantly, the absorption of FL in the gastrointestinal tract seems to depend upon the free or bound form of this compound [53]. When free FL was administered to rats and humans, around 60% was found in urine. Contrarily, when FL was administered in the bound form, the excretion was estimated between 3 and 10% [53,54,55,56]. Only 1–3% was excreted via feces [54]. This low excretion was reported to be due to the high microbial fermentation of this compound, which contributed to its reduced absorption [13,56,57].

##### 3-deoxyglucosone

3-deoxyglucosone(3-DG), which derives from the dehydration and rearrangement of Amadori products, presents highly reactive carbonyl groups [58]. It is found in high amounts in carbohydrate-rich processed products, e.g., syrups and honey [16]. Elevated plasma levels of 3-DG in CKD have been attributed to the loss of the reductase enzyme responsible for the catabolism of 3-DG, which is located in the kidneys [58]. However, diet might contribute to a further rise in its plasma level. This is suggested by a study by Rückriemen et al., who demonstrated that urinary excretion of 3-DG endogenous metabolites (3-deoxyfructose and 2-keto-3-deoxygluconic acid) was increased after meal consumption in healthy humans [59]. 

##### Glyoxal and Methylglyoxal

Glyoxal and methylglyoxal (MG) can derive from sugar fragmentation or lipid degradation [17,45]. Many food products, e.g. bread, boiled potatoes, honey, heated fats, and many beverages (beer, cola, and roasted coffee), as well as cigarette and air pollution, are the sources of these compounds [17,18]. MG has also been identified as a microbial-derived compound and is, therefore, present in fermented products, including alcoholic drinks and dairy products [17]. Studies pointing out the effect of diet content on the plasma levels of glyoxal and MG are controversial. In a study by Nakayama et al., the consumption of glucose-carbonated soda drinks rich in glucose and MG raised the plasma levels in the short term in healthy volunteers [60]. However, it is not clear whether this increase in the plasma levels was due to the content of MG per se or due to the glucose drink content. The latter option seems to be the most plausible one according to another study, where the administration of a test meal rich in carbohydrates led to higher levels of MG and 3-DG, probably due to the host metabolism [61]. In addition, an in vitro digestion study showed that MG is highly reactive toward lysine and arginine residues that are present in digestive enzymes/proteins along the gastrointestinal tract [62]. This property, together with the putative capacity of intestinal epithelial cells to degrade MG via the glyoxalase system, could be the reason why MG quantity absorbed in such a way may not be relevant [62]. This is in line with the finding of the same authors that rich-in-MG honey supplementation did not lead to an increase in urinary MG and its metabolite D-lactate [62]. Nonetheless, no longitudinal or metabolic studies have been conducted on CKD patients. Therefore, no conclusions can be reached regarding the potential effect of a diet enriched in glyoxal and MG on the plasma retention of patients with reduced kidney excretion capacity.

#### 3.1.2. Advanced Glycation End-Products: Pentosidine, Nε-carboxymethyllysine, and Nε-carboxyethyllysine

Pentosidine, Nε-carboxymethyllysine (CML), and Nε-carboxyethyllysine (CEL) belong to the class of compounds generated during the advanced stages of Maillard reactions. While pentosidine is solely generated as an advanced glycation product, CML and CEL are also derived from lipid peroxidation and ascorbate autoxidation.

Foods rich in AGEs include meat, especially when cooked under dry heat, higher-fat aged cheese, and high-fat spreads (butter, cream cheese, margarine, and mayonnaise) [20]. Of note, the type of lipid added in the processing determines the final AGE content of a certain food. For instance, scrambled eggs cooked using cooking spray, margarine, or oil had up to 75% fewer AGEs than if prepared with butter [20]. Foods with lower AGEs include grains, legumes, bread, vegetables, fruits, and milk, when not prepared with added fats [20].

Like most of the other Maillard reaction products, pentosidine can derive from endogenous sources and diet [63]. Förster et al. demonstrated in healthy volunteers that a diet low in AGEs leads to a decrease in pentosidine urinary levels. The authors hypothesized that pentosidine in its free form, such as that present in coffee, is absorbed more easily compared to the protein-bound form, which is present mostly in bakery products [63]. 

Nε-carboxymethyllysine (CML) and Nε-carboxyethyllysine (CEL) are among the most recognized AGEs. Studies concerning the metabolism and kinetics of CML and CEL have been carried out mostly in rodents and highlighted the accumulation of these compounds in specific organs, such as the kidney [64,65,66], suggesting the potentially detrimental effect of dietary AGEs in a uremic condition. Controversial results regarding the role of dietary AGEs on their plasma concentration were found by different authors when CML or CEL was administered to rodents [66,67,68,69]. However, none of these studies have been performed in CKD animal models. A study in human infants found that formula-fed babies had a 60% higher plasma concentration of CML, confirming the putative importance of dietary AGEs in humans [70]. 

## 4. Proteins

Dietary proteins are composed of essential and non-essential amino acids. As previously mentioned, amino acids contained in foods can react with reducing sugars to form Maillard reaction products during heat treatments. The main amino acids involved in these reactions are lysine, glycine, tryptophan, and tyrosine. Other amino acids, such as alanine, valine, leucine, isoleucine, phenylalanine, proline, methionine, asparagine, and glutamine, are considered intermediate browning-producing amino acids [71]. 

Other than dietary amino acids that are contained in foods and that are susceptible to modifications during processing and cooking, specific amino acids deserve attention as PBUT precursors. These include phenylalanine, tryptophan, tyrosine, methionine, and lysine, which are essential amino acids, and ornithine and arginine, which are non-essential amino acids.

Phenylalanine, together with tyrosine, is a known precursor of the PBUTs belonging to the classes of hippurates and phenols. Tryptophan is involved in the formation of indoles. Methionine plays a role in homocysteine plasma retention. Ornithine, lysine, and arginine lead to the microbial formation of polyamines. Of note, these amino acids are not the only precursors of these PBUTs. Other food components may lead to their formation too. Below, we discuss PBUTs that are derived from amino acids and specify which other nutrients may lead to their formation.

### 4.1. Hippurates

Hippuric acid derives only partially from phenylalanine [72]. It is mostly formed after the glycine conjugation of benzoate [73] or from the conversion of quinic acid [74], shikimic acid [75], and polyphenols, such as chlorogenic acid and (+)-catechin [37]. Hydroxyhippuric acid is generated from phenolic compounds that possess a hydroxyl group attached to their aromatic ring, e.g., caffeic acid, flavan-3-ol, cyanidin, and quercetin. [76]. Hippurate precursors are highly present in vegetables, fruit, tea, coffee, and whole grains [22,23] but are also found in foods where sodium benzoate is added as a preservative (E211), e.g., margarine, sauce, marmalade, gelatin, liqueurs, beer, fruit juice, and soft drinks [24]. A high intake of benzoic acid is associated with an increased production of hippurate, which, in physiological conditions, is excreted in urine [77,78]. A high urinary hippurate concentration has been used in several studies as a biomarker for fruit and vegetable intake, and it is generally associated with a positive outcome [79,80]. A recent study in CKD patients found that a dietary acid load corresponding to a high intake of animal-derived food was inversely associated with hippurate plasma concentration. However, higher hippurate plasma concentration was not associated with an increased risk of developing CKD [81]. 

The metabolism of hippurate seems to occur mostly endogenously in the mitochondrial matrix [82]. Two reactions are responsible for its formation, the first one converting benzoic acid to benzoyl adenylate and then to benzoyl-CoA, and the second one requiring the entrance of glycine in the mitochondria and its reaction with benzoyl-CoA to give hippurate [22]. It has been shown that both glycine and CoA are essential for the formation of hippurate [83,84,85]. Interestingly, while hippurate is mostly endogenously produced, the bioavailability of polyphenols and benzoate in foods is modulated by the enteric microbiome [86,87]. The role of the gut microbiome was investigated by comparing the serum concentrations of different uremic-retention solute concentrations in hemodialysis patients with and without a colon. Both p-cresyl sulfate and indoxyl sulfate were almost completely absent in patients without a colon, indicating that the colon is the sole source of these metabolites. A non-significant lower retention of hippurates was noted in subjects without a colon [88]. However, a few years later, the same samples were analyzed by LC-MS, and hydroxyhippuric acid was found to be a colon-derived solute [89]. Of note, colectomy patients may also present an increased capacity for the absorption of polyphenols in the small intestine, as suggested in a study in ileostomy patients by Khale et al. [90].

To conclude, so far, no dietary intervention studies have been carried out in CKD patients to evaluate the impact of a diet rich in benzoate and derivatives. However, we may hypothesize that a diet low in glycine may contribute to a reduction of its conversion into hippuric acid. At the same time, a modulation of the microbiota could reduce the bioavailability of hippurate precursors. 

### 4.2. Indoles

Tryptophan is the precursor of all the indoles considered uremic toxins, i.e., kynurenine, kynurenic acid, melatonin, quinolinic acid, indoxyl sulfate, and indole-3-acetic acid [8,11]. Tryptophan is an essential amino acid that is highly present in several protein-rich foods, e.g., poultry meat, red meat, pork, tofu, fish, beans, milk, nuts, seeds, oatmeal, and eggs [28]. While most tryptophan is absorbed and enters one of the two main host metabolism pathways, i.e. the kynurenine and serotonin pathways, a minor percentage of tryptophan escaping digestion reaches the colon, where it is metabolized into indole derivatives by the gut microbiome, and this has been known for more than 80 years [91,92]. 

#### 4.2.1. Uremic Toxins Generated via the Kynurenine Pathway

Most of the tryptophan ingested is degraded via the kynurenine pathway to kynurenine, kynurenic acid, quinolinic acid, picolinic acid, and nicotinamide adenine dinucleotide (NAD). The key enzymes necessary for tryptophan conversion into formylkynurenine and then into kynurenine are tryptophan 2,3-dioxygenase (TDO), which is located in the liver, and indoleamine 2,3-dioxygenase (IDO) found in extrahepatic tissues [93].

Kynurenine can be further metabolized into anthranilic acid, kynurenic acid, or 3-hydroxy kynurenine [94]. Anthranilic acid can be converted into quinolinic acid after a two-step enzymatic reaction [94]. Quinolinic acid is the precursor of NAD+, which is a cofactor for ATP synthesis [94].

Tryptophan metabolites have been reported to be involved in inflammatory processes [95]. Specifically, the kynurenine pathway is hyperactivated in chronic inflammatory conditions in an attempt to induce immune tolerance [95]. That was also highlighted by studies in CKD rats, which demonstrated that liver TDO is overexpressed and may contribute to a further increase in the retention of kynurenine [96]. Diet may represent an additional factor. Studies performed in the 1980s–1990s showed that oral and intravenous administration of tryptophan leads to a short-term increase in kynurenine and serotonin blood concentration. In physiological conditions, serum concentrations normalize after about eight hours [97,98]. 

Interestingly, not only humans but also yeasts and bacteria can metabolize tryptophan via the kynurenine pathway [27]. Kynurenine and kynurenic acid are present in fermented food products. Foods rich in kynurenine and kynurenic acid include dairy products, beer, wine, bread, honey, broccoli, and potatoes [27,29]. Boiling vegetables, such as broccoli and carrot, in water reduces their kynurenic acid content [29]. Absorption of food-derived kynurenic acid has been demonstrated [29]. However, no studies have quantified the dietary contribution and the endogenous formation of kynurenine and kynurenic acid due to the inflammatory status in a condition of CKD. 

Lastly, studies in healthy rats found that serum concentration of quinolinic acid can be reduced by a low dietary protein intake [99,100]. Fukuwatari et al. confuted the hypothesis that an increased retention of quinolinic acid could mostly be due to the impaired activity of the kidney. The authors induced CKD in rats using an adenine supplementation model and observed a more important role of the liver in the tryptophan metabolism into quinolinic acid compared to the kidney [101,102].

#### 4.2.2. Uremic Toxins Generated via The Serotonin Pathway

Melatonin is a hormone produced by the pineal gland and enterochromaffin cells responsible for the circadian rhythm [103]. In a CKD condition, it is classified as a uremic retention solute. Melatonin derives from the serotonin pathway. A very small part of dietary tryptophan is converted via this pathway into serotonin and, subsequently, into melatonin in the pineal gland via a two-step enzymatic reaction [104]. A crossover dietary intervention trial examined metabolites derived from a whole grain dietary pattern compared to those derived from an added-in-sugar diet in healthy volunteers [105]. The authors found a lower level of melatonin when the whole grain diet was followed. These and other findings indicate that diet may influence tryptophan metabolism in general and melatonin production specifically [105]. 

Moreover, melatonin is found in foods, such as eggs, fish, nuts, cereals, seeds [31], wine [106], and beer [107]. In addition, researchers have found that baking bread lowers cereals’ and seeds’ content of melatonin [31]. Some years ago, it has been demonstrated that dietary melatonin is a bioavailable compound that can be found in plasma after ingestion and in urine in the form of its metabolite sulfatoxymelatonin [107,108,109]. Despite that, no data are available on CKD.

#### 4.2.3. Uremic Toxins Generated via Gut Microbial Fermentation of Tryptophan

##### Indoxyl Sulfate

Indoxyl sulfate is one of the most studied uremic toxins. It represents the main product of gut bacterial fermentation of the amino acid tryptophan. Tryptophan is converted via the tryptophanase enzyme mostly into indole [91], which is then converted into indoxyl sulfate in the human liver [110].Studies in the 1960s corroborated that an increase in dietary tryptophan intake leads to an increase in indoxyl sulfate production [111]. Specifically, Bryan noted that 3% of orally supplemented L-tryptophan was found in urine as indoxyl sulfate in healthy volunteers. Interestingly, when tryptophan was administered directly in the ileum, the percentage of its conversion into indoxyl sulfate was raised to 14%, confirming that most of the tryptophan ingested orally might be absorbed in the small intestine and that diet contributes significantly to indoxyl sulfate generation. 

Plasma retention of indoxyl sulfate seems to be regulated by amino acid intake. A recent study in CKD mice found a reduction in indoxyl sulfate plasma retention when a low-protein diet was administered and even a further reduction when the low-protein diet was low in aromatic amino acids, namely tyrosine, tryptophan, and phenylalanine [112].

Another recent study in CKD mice highlighted the importance of sulfur-containing amino acids, cysteine, and methionine [113]. A diet enriched with sulfur amino acids resulted in lower serum concentrations of indoxyl sulfate. Lower indole production at the gut level was hypothesized to be the consequence of the capability of sulfuric amino acid metabolites to inhibit the microbial tryptophanase enzyme [113]. Of note, in both studies, the authors found an improvement in kidney fibrosis, a clear sign that the modulation of diet has a direct effect on uremic toxicity in CKD [112]. 

Another remarkable food intervention study in CKD mice found that the supplementation of (+)-Sesamin, a sesame lignan, inhibits the enzyme converting tryptophan into indole in gut bacteria, suggesting the potential effectiveness of encapsulated (+)-Sesamin to reach the colon and exert a beneficial effect in CKD [114].

In humans, few dietary intervention studies attempted to decrease indoxyl sulfate plasma levels. Poesen et al. demonstrated that dietary proteins influenced endogenous and microbial tryptophan [115]. Few studies investigated the impact of a low-protein diet. In a 6-months trial, a low-protein diet did not lead to significant differences in indoxyl sulfate but in p-cresyl sulfate only [116]. Marzocco et al. found that a very-low-protein diet with 0.3 g/kg body weight was more effective than a low-protein diet (0.6 g/kg body weight) in reducing indoxyl sulfate plasma concentration [117]. In another study plasma indoxyl sulfate and p-cresyl sulfate were associated with the protein–fiber ratioconsumed, highlighting that both diet and gut microbial diversity affect plasma retention [118]. In this regard, Sirich et al. found that a higher dietary fiber intake in the form of resistant starch in hemodialysis patients reduced plasma indoxyl sulfate [119]. 

Studies carried out to modulate the gut microbiome have produced controversial results. A study making use of probiotics, namely a mix of *Streptococcus thermophilus*, *Lactobacillus acidophilus*, and *Bifidobacteria longum*, in CKD patients was not only ineffective but even significantly increased indoxyl sulfate plasma concentration in CKD patients [120]. That suggests that caution is needed when prescribing probiotics to CKD patients. Inconclusive results have been reached in studies testing synbiotics, i.e., rich in pre- and probiotics. A study conducted on 58 CKD patients in Brazil showed that a 6-week administration of a synbiotic meal, in this case, 100 mL of probiotic dairy drink with *Bifidobacterium longum BL-G30* and extruded sorghum flakes, led to a decrease in indoxyl sulfate, p-cresyl sulfate, and indole-3-acetic acid plasma retention compared to their levels before the intervention [121]. A very recent study conducted in Egypt reported similar positive results on serum indoxyl sulfate after administering to hemodialysis patients yogurt containing five strains of *Lactobacillus* and *Bifidobacterium* together with lactulose syrup for six weeks [122]. Another study evaluated the effect of synbiotics (*Lactobacillus acidophilus*, *Bifdobacterium longum*, and fructooligosaccharides (FOS)) for 60 days in 57 CKD patients, finding no effect on indoxyl sulfate [123]. Other human interventional studies providing synbiotics have attempted to lower indoxyl sulfate but have found no or even detrimental effects [124,125]. Such a high heterogeneity of the results derive from the use of different probiotic strains, prebiotics of various origins, diverse structures, and different matrices of foods in interventions including biotics. Moreover, contrasting conclusions can be explained by differences in patient characteristics, study sample size, renal function status, and follow-up time of the clinical trials. Nonetheless, in summary, dietary interventions may modulate indoxyl sulfate production.

##### Indole-3-acetic Acid

Indole-3-acetic acid is generated mostly by gut bacteria that are able to convert tryptophan [126,127]. Specifically, five bacterial precursors are involved in indole-3-acetic acid formation: indole-3-pyruvic acid, tryptamine, indole-3-acetamine, indole-3-acetonitrile, and tryptophan, with the first two accounting for the major part of the contribution [128]. The mostly microbial origin was recently confirmed in a study in rats that received antibiotics, showing very low levels of indole-3-acetic acid [129]. 

Few studies examined how diet influences the levels of indole-3-acetic acid produced. As early as in 1959, it was discovered that a load of tryptophan provided orally to healthy humans can lead to a post-prandial increase in urinary indole-3-acetic acid [130]. In 1965, Sprince et al. found that a diet enriched in methionine but lacking in nicotinic acid, also called vitamin B3, leads to an increase in urinary indole-acetic acid in rats and suggested that methionine favors the conversion of tryptophan into indole-3-acetic acid [131]. Recent studies highlighted the role of the gut microbiome–diet interlink. An inverse correlation was found between serum-free indole-3-acetic acid and free indoxyl sulfate with fiber consumption in children with CKD [132]. However, a supplementation of resistant starch, a type of fiber, to CKD patients on hemodialysis did not lead to a decrease in the indole-3-acetic acid plasma level [133].Of note, indole-3-acetic acid was recently shown to be produced by the host, specifically in immune cells possessing the interleukin-4-induced gene 1 (IL4I1), which encodes a protein that converts tryptophan, phenylalanine, and tyrosine [134]. It remains unclear to which extent the host production contributes to the plasma level.

Lastly, indole-3-acetic acid is a known plant hormone that is present in plant foods, such as cereal grains [32]. However, no studies have been performed to assess its impact on plasma retention.

### 4.3. Phenols

Phenylalanine, tyrosine, and polyphenols are the main precursors of phenol toxins, which include 2-methoxyresorcinol, phenol, hydroquinone, p-cresyl sulfate, p-cresyl glucuronide, and phenylacetic acid. 

#### 4.3.1. 2-methoxyresorcinol

2-methoxyresorcinol is a polyphenol that derives from the consumption of green and black tea [33,135]. Urinary 2-methoxyresorcinol was identified as a biomarker of tea consumption [33]. However, no studies have been carried out to elucidate its bioavailability and contribution to plasma retention in humans.

#### 4.3.2. Phenol and Hydroquinone

Phenol and hydroquinone derive from the ingestion of arbutin-containing foods, the catabolism of tyrosine and phenylalanine by gut bacteria, the use of specific medicines, the smoking habit, and the environmental exposures to benzene [34]. Diet-derived phenols are especially present in certain foods, such as coffee, tea, plant-based products, alcoholic beverages, dairy products, fruits, roasted nuts, honey, molasses, beef, and spices [34]. On the other hand, arbutin is a glucose conjugate of hydroquinone that undergoes hydrolysis in the stomach and turns into free hydroquinone, which is then absorbed along the gastrointestinal system. Arbutin is naturally found in foods, such as wheat germ and pears, and to a lower extent in beverages, such as coffee, tea, and red wine [34]. Deisinger et al. assessed whether dietary hydroquinone affected plasma and urinary concentrations. In healthy volunteers, the authors found that phenol concentration was increased after ingestion of a high-hydroquinone meal when compared to a low-content meal [136]. In contrast, no similar studies have been reported in CKD patients. Simple phenols mainly originate from gut fermentation of tyrosine and plant phenolics in the distal colon. Tyrosine is provided by the diet or by the hydroxylation of phenylalanine [137]. 

#### 4.3.3. P-Cresyl Sulfate and P-Cresyl Glucuronide

P-cresyl sulfate and p-cresyl glucuronide are gut microbial metabolites derived from the conversion of dietary tyrosine. Specifically, a small amount of this amino acid that escapes digestion and arrives in the colon is converted to p-cresol by gut bacteria. After p-cresol is absorbed, it is sulfated by hepatocytes [138] to become p-cresyl sulfate or glucuronidated by liver and kidney enzymes [139] to become p-cresyl glucuronide.

Similar to indoxyl sulfate, a modulation of the plasma retention of these metabolites may be possible via the diet and microbiome modifications. A recent study showed that higher tyrosine and phenylalanine content in diet contributes to p-cresyl sulfate plasma levels in non-dialyzed patients with CKD after correcting for GFR [140]. 

A recent randomized double-blind human study in hemodialysis patients showed that supplementation of curcumin can decrease plasma p-cresyl sulfate concentrations, whereas concentrations of indoxyl sulfate and indole-acetic acid are not significantly altered [141]. According to the authors, this may be due to a decrease in p-cresol-producing gut bacteria, such as those belonging to *Prevotellaceae* species [141]. A potentially similar reason may explain the results of Salmean et al., who found that daily administration of muffins enriched with fibers (pea hull fiber and inulin) lowered the plasma levels of p-cresol by 24% in CKD patients [142]. Similar results were obtained by administering oligofructose-inulin to hemodialysis patients by our group [143].

#### 4.3.4. Phenylacetic Acid

Phenylacetic acid is another amino acid-derived uremic toxin. It mostly derives from the gut microbial metabolization of phenylalanine and tyrosine [144]. Around 0.4% of phenylalanine and 0.1% of tyrosine ingested can be found in urine as phenylacetic acid in humans [145]. Studies in healthy and uremic subjects showed that a phenylalanine load after overnight fasting led to a higher increase in the plasma concentrations of phenylalanine and conjugated phenylacetic acid in uremic patients, but not in the free form of phenylacetic acid [146]. Despite this long-term study, phenylalanine intake did not lead to increased phenylacetic acid plasma retention in healthy volunteers [147]. Conclusions cannot be drawn for CKD patients, who seem to also suffer from a disbalance in phenylalanine/tyrosine conversion and metabolism [146].

Lastly, foods may also contain phenylacetic acid. It has been reported that phenylalanine may be degraded when products of lipid peroxidation are also present, leading to phenylacetic acid formation [148]. Though, it is not known to which extent this phenylacetic acid present in foods can be absorbed and contribute to plasma retention.

### 4.4. Polyamines

Polyamines that are found to be potentially toxic in CKD are putrescine, spermidine, and spermine. Mammalian cells can contribute to their biosynthesis via a four-step reaction, starting from the amino acid arginine [149]. Arginine is converted into L-ornithine and, from here, to putrescine via a reaction with S-adenosyl methionine. Spermidine is produced starting from putrescine thanks to the spermidine synthase enzyme. Spermine derives from spermidine conversion [149]. Despite the endogenous production, the intestinal content of putrescine and spermidine is mainly due to gut microbes, differently from spermine [150]. Microbiome-derived polyamines stem from the catabolism of dietary ornithine, lysine, and arginine [149]. Arginine, which is also the precursor of ornithine, is particularly present in animal-derived foods but also in nuts and soybeans [41]. However, no human studies have shown an association between dietary arginine and polyamine plasma retention. The modulation of a gut microbiome dysbiosis condition was suggested as a potential mechanism in a recent CKD rat study, where animals treated with Poria cocos, a medicinal fungus, showed a significant reduction in polyamine retention. Thus, gut microbiome modulation plays an important role and may be addressed to reduce polyamine accumulation [151].

In addition, polyamines can also be found in several foods. Fruit, corn, sweet potatoes, and cheese contain high levels of putrescine. Vegetables, corn, and soybeans possess high levels of spermidine. Meat products are rich in spermine [41,149]. Of note, vegetables can leach up to 40% of their polyamine content when boiled in water, and roasting coffee lowers its polyamine level [152]. Intriguingly, meat storage increases the level of polyamines probably because of bacterial presence, while fish storage generally leads to a higher amount of putrescine but not spermine and spermidine [152].

Dietary polyamines are absorbed along the gastrointestinal tract [153]. Few interventional studies have been carried out to assess the impact of dietary polyamines on their plasma levels. In a study in mice and humans, Soda et al. found that 26 weeks of a high-polyamine diet were needed to significantly increase the plasma levels of spermine and spermidine in mice [154]. On the other hand, a diet enriched in natto, a Japanese soybean product with a high content of polyamines, led to an increased plasma level of spermine in healthy humans [154]. However, in another study in older adults, a supplementation of polyamine capsules did not lead to differences in spermidine plasma concentration [155]. Of note, foods naturally enriched in specific components may possess a different bioavailability and absorption compared to capsules. For this reason, conclusive observations cannot be formulated. Moreover, no studies on reduced or increased intake of dietary polyamines have been conducted in CKD patients.

### 4.5. Homocysteine

Homocysteine is a uremic toxin correlated with increased cardiovascular risk that is derived from a dietary amino acid, methionine. Methionine is converted first into S-adenosylmethionine and consequently demethylated to S-adenosylhomocysteine, a precursor of homocysteine [156]. Folates (vitamin B9) and cobalamin (vitamin B12) are involved in homocysteine metabolism, preventing its formation and favoring its re-methylation to methionine [157]. Another pathway for this reaction uses betaine as a source of methyl groups. Pyridoxin (vitamin B6) is not only important in folate metabolism, but it also plays a role in the conversion of homocysteine to cysteine [158]. 

The etiology of hyperhomocysteinemia in CKD patients is controversial. Existing hypotheses include accumulation due to decreased clearance, disbalance in folate metabolism, augmented levels of homocysteine precursors, and impaired function of the enzymes involved in its metabolism [158]. In healthy humans, oral methionine loading resulted in an increased homocysteine concentration [159]. However, Haulrik et al. found no effect of a high-methionine diet for six months on homocysteine levels in overweight adults [160]. Studies have also been performed to assess the effect of the supplementation of vitamin B6, B12, and folates on homocysteine levels in CKD patients. A study in hemodialysis patients found no improvement in homocysteine levels after a 4-week supplementation of different dosages of folic acid [161]. Similarly, Wrone et al. noted no benefit of folates on cardiovascular event incidence in end-stage renal disease patients [162]. In end-stage kidney disease patients with low levels of vitamin B12, its supplementation via intravenous injection for four weeks led to a 35% reduction in homocysteine plasma levels [163]. Similar positive results have been more recently reported in a 2-month randomized controlled trial in hemodialysis patients [164]. Of note, the prescription of vitamin B12 should be cautious. High dosages of vitamin B12 might be detrimental in CKD because of the formation of the toxic compound cyanide, which is eliminated via the kidneys in physiological conditions [165]. Lindner et al. investigated the effect of the supplementation of vitamin B6, and vitamin B6 together with folate, in hemodialysis and end-stage renal disease patients. They found lower homocysteine levels only in the hemodialysis group. Here, vitamin B6 alone contributed to a modest decrease in homocysteine level, while vitamin B6 with folic acid together led to a more pronounced decrease [166]. The Heart Outcomes Prevention Evaluation (HOPE)-2 study, which included 3310 patients, revealed that a concomitant supplementation of these three vitamins for five years in CKD patients helped lower the levels of homocysteine in CKD patients on treatment compared to those on placebo [167]. However, no improvement in other outcomes, such as cardiovascular death, was observed [167].

## 5. Fatty Acids

Dietary fatty acids can be classified into three main types: saturated fatty acids, monounsaturated fatty acids (MUFAs), and polyunsaturated fatty acids (PUFAs). Among the PUFAs, omega 3 and omega 6 are the most studied fatty acids in terms of health effects. In addition to those, uncommon fatty acids include furan fatty acids and conjugated fatty acids, which are present in a minor concentration in foods [42]. Furan fatty acids are fatty acids possessing a furan ring in their chemical structure. Despite being less common in foods, their role in cardiometabolic health has been highlighted [42]. Although chronic kidney disease research shows a limited impact of dietary lipids on the plasma retention of uremic toxins, furan fatty acids and long-chain omega 3 fatty acids have been identified as the precursors of CMPF, another PBUT.

### 3-Carboxy-4-methyl-5-propyl-2-furanpropionate (CMPF)

3-Carboxy-4-methyl-5-propyl-2-furanpropionate (CMPF) originates from the endogenous metabolism of furan fatty acids and long-chain omega 3 fatty acids mostly found in fish and derivatives [168]. Interestingly, fish oil supplement capsules contain a lower amount of furan fatty acids because of their elimination during capsule production [169]. Furan fatty acids are also present in minor quantities in green plants, soybean oil, and butter [169]. Studies showing an association between fish oil intake and CMPF biological fluids concentration started in the 1990s. Wahl et al. found a 3-time increase in the plasma level of this metabolite and a 5–6-time increase in its urinary excretion after an administration of four weeks of fish oil [170]. Increases in CMPF plasma levels have also been observed in diabetic patients [171] and overweight subjects [172]. It was calculated that CMPF accounts for approximately 0.4% of plasma levels of long-chain omega-3 fatty acids [168]. 

To our knowledge, it cannot be excluded that furan fatty acids may derive also from the gut microbiome. Indeed, they are produced by some bacterial strains, which are also known to be present in our intestines. However, studies quantifying them and showing the contribution of the gut microbiome to CMPF plasma concentration are lacking [173].

Overall, despite the absence of data concerning uremic patients, CMPF is considered a biomarker of fish intake, and fish consumption likely contributes to the plasma accumulation of this highly protein-bound metabolite.

## 6. Micronutrients

### Polyphenols

As previously mentioned, some polyphenols may be considered PBUT precursors. Because of their scarce bioavailability, many of these compounds can be fermented by the gut microbiome [174]. Hippurates can indeed derive from the conversion of chlorogenic acid and (+)-catechin but also from phenolic compounds, e.g., caffeic acid, flavan-3-ol, cyanidin, and quercetin. 

2-methoxyresorcinol is also a polyphenol introduced into the body via the diet. Similarly, other phenols considered to be uremic toxins can be generated after the ingestion of food-derived phenolic acids. However, the intake of fruits and vegetables, the main source of these micronutrients, was not found to be associated with plasma levels of p-cresyl sulfate in hemodialysis patients in a recent cross-sectional study conducted in Australia [175].

Overall, due to a lack of studies showing the direct influence of polyphenol intake on uremic toxicity and considering their protective role as antioxidants, polyphenols should not be excluded from diet in CKD. 

## 7. Future Recommendations

Further long-term studies and nutritional interventions are required to determine the impact of diet composition on the accumulation of protein-bound uremic retention solutes in chronic kidney disease. Longitudinal studies in CKD patients are lacking in the literature. Despite an increase in uremic toxins expected over time, these studies may help find the associations between dietary habits and lower uremic toxin plasma levels, suggesting a slowing down of disease progression. At the same time, nutritional intervention studies are needed to assess the contribution of a single or repeated administration of specific foods or nutrients to plasma retention of uremic toxins in CKD patients. It is also noteworthy that the form and the matrix of food or nutrient administered play a role in absorption and metabolism. 

Simultaneously, animal and in vitro studies are still seeking to understand the mechanisms of action of dietary components at a biological level. Concerning the latter ones specifically, organoids, multi-organ systems, and organ-on-a-chip models that are recently in development are progressing and may one day be able to more closely represent the reality of the human body metabolism.

## 8. Conclusions 

The current review summarizes findings on the role of diet as a source of uremic retention solutes and uremic toxins, or their precursors, in chronic kidney disease, focusing on protein-bound uremic toxins, their metabolism, and dietary strategies reported in the literature that have shown to be promising in reducing their plasma retention. 

There are still controversies regarding the contribution of the diet to the accumulation of plasma retention solutes in CKD, considering the derangement of metabolism occurring in CKD. In vitro and in vivo animal studies have helped identify beneficial or detrimental compounds in CKD. To date, human intervention studies that focus on plasma retention solutes as the primary or secondary endpoint are scanty, their sample size is often low, and the study endpoints are not standardized. Based on the available data, several clear conclusions can be drawn. 

A lower intake of proteins accompanied by a higher intake of fibers seems to favor a beneficial microbiome and slow down the progression of renal failure. A low-protein diet, which is low in poultry, red meat, and eggs, along with a higher consumption of polyphenol-rich foods, such as fruit and vegetables, allows a reduction in the intake of tryptophan, tyrosine, and phenylalanine, which are the known precursors of indolic and phenolic uremic toxins; the intake of glycine, which is essential for the formation of hippurates; and the intake of furan fatty acids, which are the precursors of CMPF [176]. It is noteworthy that protein restriction has also been recognized as an effective and safe treatment in the elderly population with CKD as long as patients maintain an adequate calorie intake. This is to prevent muscle wasting and sarcopenia [177]. 

The type of cooking also has a relevant impact on the content of uremic toxin precursors in foods. The co-presence of reducing sugars and amino acids in foods is what leads to the formation of IGPs and AGEs in foods when they are heated up. For this reason, precautions need to be taken both when selecting foods at the supermarkets and when cooking foods. Consumption of processed and ultra-processed foods, e.g., pasteurized and ultra-heat treatment (UHT) foods and products along with sugars, syrups, molasses and additives, such as benzoic acid, should be limited in patients with CKD. When preparing food at home, it is important to remove the broth when boiling foods rich in kynurenic acid and polyamines. Of note, this is also a method to reduce a high mineral load provided by foods, e.g., vegetables, that is also recommended in CKD [176]. In addition, fewer fats and fatspreferably of vegetable origins in the food preparation process translates into reduced formation of phenylacetic acid and AGEs in certain foods. Tea and roasted coffee may also be avoided in CKD because of their high contents of phenols, hydroquinone, and AGEs. For the same reason, the consumption of alcoholic beverages, such as beer and wine, should be discouraged, apart from their alcohol content. These considerations are in line with the nutritional guidelines for these patients [176].

## Figures and Tables

**Table 1:** List of protein-bound uremic toxins and their dietary origin. PBUT = protein-bound uremic toxin; UHT = ultra-heat treatment.

PBUT Class	Uremic Toxin	Dietary Origin	Metabolite Source	Foods Containing High Levels of PBUT	Foods Containing High Levels of Precursors
**Maillard reaction products**	Fructoselysine	Reducing sugars and amino acids	Food and host metabolism	Pasteurized milk, pasta, chocolate, cereals, and carbonated soft drinks [13]	Starchy foods, fruit, and milk [14,15]
	3-Deoxyglucosone			Carbohydrate-rich processed products, e.g., syrups and honey [16]	
	Glyoxal			Bread, boiled potatoes, honey, heated fats, and beverages (beer, cola, and roasted coffee) [17,18]	
	Methylglyoxal				
	Nε-carboxymethyllysine (CML)	Reducing sugars and amino acids, lipids, and ascorbate		Meat and fish cooked under dry heat, bakery products, cereals, and chocolate snacks [19,20]	
	Nε-carboxyethyllysine (CEL)				
	Pentosidine	Reducing sugars and amino acids		UHT milk products and bakery products [21]	
**Hippurates**	Hippuric acid	Benzoic acid, phenylalanine, quinic acid, shikimic acid, and polyphenols	Microbial metabolism	-	Vegetables, fruit, tea, coffee, whole grains [22,23], margarine, sauce, marmalade, gelatin, liqueurs, beer, fruit juice, and soft drinks [24]
	Hydroxyhippuric acid			-	
**Indoles**	Kynurenine	Tryptophan	Food and host metabolism	Fresh milk [25], breast milk, infant formula [26], beer, and dairy fermented products [27]	Poultry meat, red meat, pork, tofu, fish, beans, dairy products, nuts, seeds, oatmeal, and eggs [28]
	Kynurenic acid			Multiflorous honey, fresh broccoli, potatoes [29,30], beer, and red wine [27]	
	Melatonin			Eggs, fish, nuts, some cereals, and germinated beans [31]	
	Quinolinic acid		Host metabolism	-	
	Indoxyl sulfate		Microbial and host metabolism	-	
	Indole-3-acetic acid		Host, food, and microbial metabolism	Cereals and legumes [32]	
**Phenols**	2-methoxyresorcinol	Polyphenol	Food	Coffee and tea [33]	-
	Phenol	Tyrosine and phenylalanine and arbutin	Food and microbial metabolism	Coffee, tea, plant-based products, alcoholic beverages, dairy products, fruits, roasted nuts, honey, molasses, beef, and spices [34]	Arbutin-rich foods, such as wheat germ, and pears, and beverages, such as coffee, tea, and red wine [34].
	Hydroquinone		Food and host metabolism		
	P-cresyl sulfate	Tyrosine	Microbial and host metabolism	-	Meat and dairy products [35]
	P-cresyl glucuronide				
	Phenylacetic acid	Polyphenols and phenylalanine	Food, microbial and host metabolism	Fermented beans [36]	Fruit, vegetables, black tea [37,38]
**Polyamines**	Putrescine	Arginine and L-ornithine	Food, microbial, and host metabolism	Cereals, legumes, soy derivatives, mushrooms, peas, hazelnuts, pistachios, spinach, broccoli, cauliflower, green beans, and meat and derivatives [39]	Seafood, watermelon juice, nuts, seeds, algae, meats, rice protein concentrate, and soy protein isolate [40,41]
	Spermidine		Food, microbial, and host metabolism		
	Spermine		Food, microbial, and host metabolism		
**Others**	3-Carboxy-4-methyl-5-propyl-2-furanpropionate (CMPF)	Furan fatty acids and omega 3 fatty acids	Host and microbial (?) metabolism	-	Fish, plants, algae, and crustaceans [42]
	Homocysteine	Methionine	Host metabolism	-	Eggs, fish, and some meats [43]

## Data Availability

Not applicable.
